# Accuracy and Reliability of a Novel Method for Fusion of Digital Dental Casts and Cone Beam Computed Tomography Scans

**DOI:** 10.1371/journal.pone.0059130

**Published:** 2013-03-20

**Authors:** Frits A. Rangel, Thomas J. J. Maal, Ewald M. Bronkhorst, K. Hero Breuning, Jan G. J. H. Schols, Stefaan J. Bergé, Anne Marie Kuijpers-Jagtman

**Affiliations:** 1 Department of Orthodontics and Craniofacial Biology, Radboud University Nijmegen Medical Centre, Nijmegen, The Netherlands; 2 3D Facial Imaging Research Group Nijmegen–Bruges, Radboud University Nijmegen Medical Centre, Nijmegen, The Netherlands; 3 Department of Oral and Craniomaxillofacial Surgery, Radboud University Nijmegen Medical Centre, Nijmegen, The Netherlands; 4 Department of Preventive and Restorative Dentistry, Radboud University Nijmegen Medical Centre, Nijmegen, The Netherlands; University of Toronto, Canada

## Abstract

Several methods have been proposed to integrate digital models into Cone Beam Computed Tomography scans. Since all these methods have some drawbacks such as radiation exposure, soft tissue deformation and time-consuming digital handling processes, we propose a new method to integrate digital dental casts into Cone Beam Computed Tomography scans. Plaster casts of 10 patients were randomly selected and 5 titanium markers were glued to the upper and lower plaster cast. The plaster models were scanned, impressions were taken from the plaster models and the impressions were also scanned. Linear measurements were performed on all three models, to assess accuracy and reproducibility. Besides that, matching of the scanned plaster models and scanned impressions was done, to assess the accuracy of the matching procedure. Results show that all measurement errors are smaller than 0.2 mm, and that 81% is smaller than 0.1 mm. Matching of the scanned plaster casts and scanned impressions show a mean error between the two surfaces of the upper arch of 0.14 mm and for the lower arch of 0.18 mm. The time needed for reconstructing the CBCT scans to a digital patient, where the impressions are integrated into the CBCT scan of the patient takes about 15 minutes, with little variance between patients. In conclusion, we can state that this new method is a reliable method to integrate digital dental casts into CBCT scans. As far as radiation exposure, soft tissue deformation and digital handling processes are concerned, it is a significant improvement compared to the previously published methods.

## Introduction

Now that Cone Beam Computed Tomography (CBCT) is a regular diagnostic tool in oral- and maxillofacial surgery, clinical interests are shifting to digital planning and eventually robotic surgery for orthognathic cases. With 3D planning software surgery can be planned digitally and then transferred to the patient. However, 3D virtual planning of orthognathic surgery still suffers from the disadvantages CBCT imaging has. For orthognathic surgery a good representation of the dental surfaces as well as the occlusion is needed for the 3D planning to properly position the jaws and to reach a stable occlusion. In CBCT imaging visualization of the dentition is still difficult. Loubelle and co-workers and Schulze and co-workers showed that the density of enamel is so high, that it gives rise to artefacts around the teeth [1], [2]. Besides that, brackets will always be present in orthognathic patients, since treatment continues into the postsurgical phase. This means that the dentition is poorly visible on CBCT scans of these patients, since scattering (from the brackets) and other artefacts occur at the occlusal level [3], [4]. Integration of digital dental casts into CBCT scans, could increase the accuracy of the orthognathic procedure.

Several methods have been proposed to integrate digital dental casts in CBCT scans. Some researchers [5], [6] proposed a method, based on a best fit matching of scanned plaster models with the dentition in the CBCT scans, after removal of streak artefacts due to amalgam restorations. In these studies average distances, between the CBCT scans and the scanned plaster models were within 0.6 mm. However, in orthognathic patients, brackets are present in the upper and lower arch, resulting in streak artefacts. Cleaning these artefacts on CBCT images will result in removing all occlusal surfaces and most of the dentition, resulting in an image that lacks information to perform an accurate surface matching procedure.

Other researchers [3], [4], [7] proposed a method using bite jigs, with fiducial markers attached to it. The patient wears the bite jig when the CBCT scan is made and afterwards the bite jig is scanned together with the impressions. After data processing, the fiducial markers are visualized on both the CBCT scan and the scan of the bite jig with the impressions. With dedicated software, both data sets are matched, using the fiducial markers as reference points. The disadvantage of this method is that the fiducial markers are positioned outside the mouth and are giving a distortion of the soft tissues. In this way a reliable judgement of the soft tissues of the patient at rest cannot be obtained.

Swennen et al. [8] developed a triple scan method, using an impression tray in which both the upper and lower jaw are registered. For this procedure, a high resolution CBCT scan is made of the patient in rest. Next, a low resolution CBCT scan of the patient is made, with the impression tray placed in the mouth. Finally the impression tray is scanned separately. With dedicated software, the impression scan is placed into the CBCT scan of the patient, using the impression tray in the low resolution scan as reference. The major disadvantage of this method is that two CBCT scans of the patient are needed, which gives unnecessary x-ray exposure, while the digital data handling processes are time-consuming.

To overcome previous mentioned problems, we propose a new method to integrate digital dental casts into CBCT scans, using fiducial markers glued to the gingiva [9]. The purpose of the present study is to evaluate the accuracy and reliability of this method.

## Materials and Methods

To simulate the method, in the present study plaster casts were used to avoid radiation exposure to patients. Plaster casts of 10 patients were randomly taken. Inclusion criteria were:

- plaster casts without any physical damage
- full complement of teeth, up to and including the first molars in both dental arches
- normal morphology of all teeth
- no visible attrition, caries, or restorations affecting the mesiodistal or bucco-lingual diameter of the crown.

**Figure 1:**
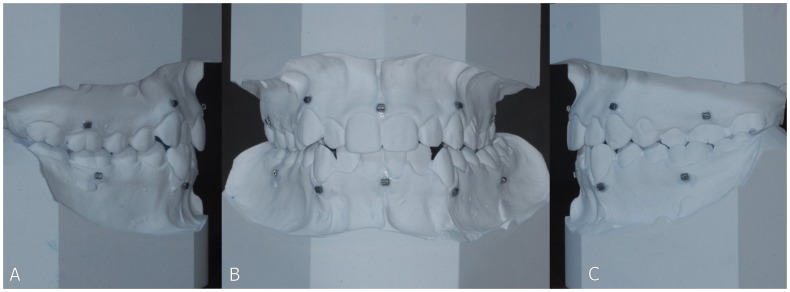
Position of the titanium markers on the physical dental cast. **A**. right view; **B**. frontal view; **C**. left view.

Titanium markers were glued (UHU Super Power, UHU GmbH & Co, Bühl, Germany) to the plaster casts: 5 to the upper plaster cast and 5 to the lower plaster cast (fig. 1). These plaster casts were scanned in occlusion, using a standardized CBCT scanning protocol (i-CAT™, Imaging Sciences International, Inc., Hatfield, USA). CBCT scanning of the plaster casts was performed in “13 cm” scan mode (field of view: 17 cm diameter, 13 cm height; scan time 40 s; voxel size 0.2 mm) at 129 kV and 47.74 mA.

After scanning of the plaster casts, impressions of the plaster casts were taken, using plastic impression trays (TP Orthodontics, Inc., La Porte, Indiana, USA) and orthodontic alginate (Cavex Orthotrace, Cavex Holland BV, Haarlem, The Netherlands). After hardening, the impressions were removed from the plaster casts with the markers embedded in the impression. All impressions were then scanned, using the same standardized CBCT scanning protocol.

All CBCT scans were exported as DICOM data sets and imported into Maxilim 2.3.0 viewing software. (Medicim NV, Mechelen, Belgium). Out of the scans of the plaster casts and the impressions, a 3D reconstruction of the dentition was made. An isosurface was extracted by thresholding the DICOM images.

**Figure 2:**
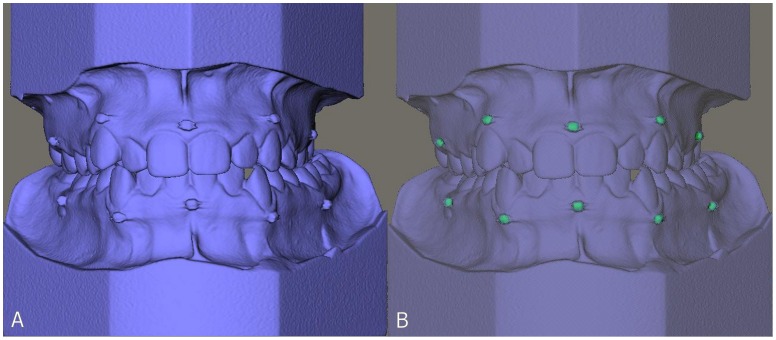
3D reconstruction of the dental cast. **A**. normal reconstruction; **B**. 3D reconstruction with markers extracted.

A calibration of the plaster casts was performed by first scanning a plaster cast with known dimensions. In this way, the correct gray values for plaster material could be determined, compensating for the beam-hardening effect [10], [11]. For the plaster casts a grey value of 1900 was chosen as limit for the extraction of the isosurfaces (fig. 2a). In a second extraction (in the same model), the markers were separately extracted, using a grey value of 3500 (fig. 2b). This reconstruction was called the scanned plaster cast.

**Figure 3:**
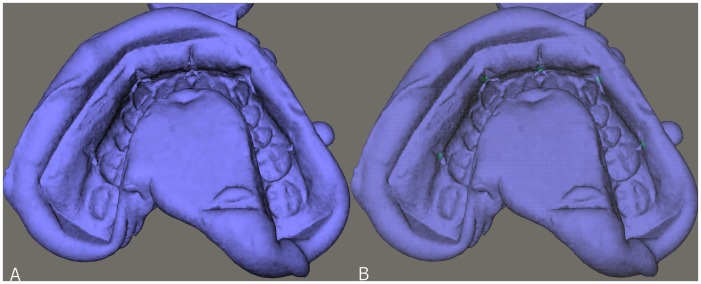
3D reconstruction of the scanned impression. **A**. normal reconstruction; **B**. 3D reconstruction with markers extracted.

Out of the DICOM data set of the impressions a 3D reconstruction was made, using a grey value of −700 (fig. 3a). In a second extraction (in the same model), the markers were separately extracted, using a grey value of 3500 (fig. 3b). This reconstruction was called the scanned impression.

**Figure 4:**
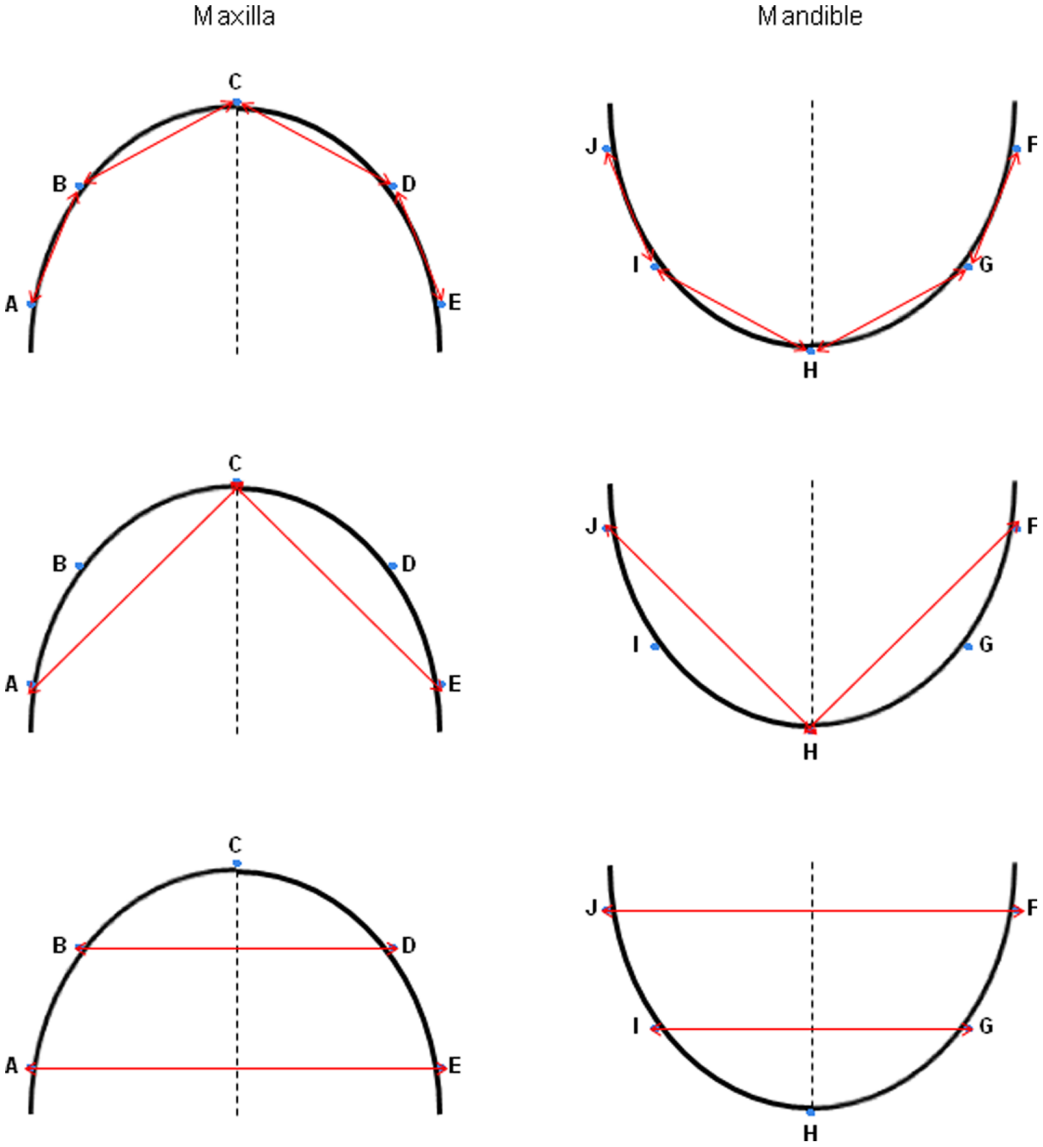
Measurements performed on all three models. A–J are markers; Distances are indicated with red arrows.

On all three models (the plaster cast, the scanned plaster cast and the scanned impression) linear measurements were made by one observer (FR). The measurements for the maxilla and mandible are shown in figure 4. Measurements on the plaster casts were made using a digital calliper (Absolute Digimatic Caliper: 500–151 U, Mitutoyo UK LTD, Andover, Hampshire, UK). Measurements on the digital models were done using dedicated software (Maxilim® 2.3.0., Medicim NV, Mechelen, Belgium). All measurements were placed in a Microsoft Excel worksheet.

To calculate the error of the method all measurements were repeated ten times, with one week in between measurements on the same model.

**Figure 5:**
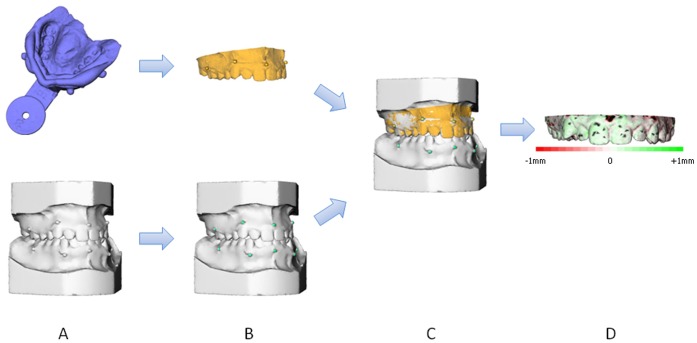
Matching procedure of the digital dental casts and the digital impressions. **A**. Original datasets: 3D reconstructed scanned plaster cast and scanned impression; **B**. Original datasets with markers extracted; **C**. Marker based registration, resulting in integration of the scanned plaster cast and scanned impression; **D**. Distance map, which shows the difference between the two surfaces.

After construction of the scanned plaster casts and the scanned impressions, the two models were fused, using dedicated software (Maxilim® 2.3.0., Medicim NV, Mechelen, Belgium). The two models were matched on the titanium markers, using a marker based registration procedure [12] (fig. 5a–d). After matching, differences between the two surfaces could be visualized as a colour histogram. This colour histogram was computed out of the differences on a large number of points (+/−25,000) (fig. 5d).The mean distance between the two surfaces was calculated and visualized in a so called distance map.

The clinical procedure and data handling was timed, using a digital stopwatch.

### Data Analysis and Statistics

Means and standard errors were calculated for all dimensions. The mean error was calculated as the standard deviation of the ten repeated measurements. This was averaged over the ten casts. To determine the reproducibility of the method, the measurement errors of the three models were compared, using a student’s t-test. To determine the accuracy two different methods were used. First the measurements made on both digital models were compared to the measurements made on the plaster cast, using the students paired t-test. Second the average distance between the surfaces was calculated from the distance map. Data analysis and statistics were performed using the Statistical Package for Social Sciences (SPSS, Chicago, IL; version 13.0).

## Results

Table 1 shows the mean measurement errors for each measurement and the p-value and 95% CI for the comparison of the measurement error between the three models. Table 2 shows the comparison between the three models.

**Table 1:** Measurement errors of the three models and comparison between the three models, for each measurement: p-value and 95% confidence interval.

	plaster cast		scanned plaster cast		scanned impression		p-value								95% Confidence Interval									
measurements	meanerror	SE oferror	meanerror	SE oferror	meanerror	SE oferror	plaster vsscannedplaster		plaster vsscannedimpression			scannedplaster vsscannedimpression			plaster vsscanned plaster				plaster vsscannedimpression			scanned plastervs scannedimpression		
A–B	0.086	0.007	0.040	0.008	0.034	0.004	**0.002**	*		**0.000**	*		0.592			[0.022	…	0.072]	[0.033	…	0.070]	[−0.016	…	0.026]
B–C	0.109	0.008	0.068	0.010	0.041	0.005	**0.011**	*		**0.000**	*		**0.037**	*		[0.012	…	0.069]	[0.045	…	0.090]	[0.002	…	0.051]
C–D	0.108	0.014	0.042	0.005	0.031	0.003	**0.002**	*		**0.000**	*		0.095			[0.032	…	0.101]	[0.045	…	0.110]	[−0.002	…	0.024]
D–E	0.107	0.012	0.032	0.003	0.044	0.007	**0.000**	*		**0.002**	*		0.169			[0.047	…	0.105]	[0.031	…	0.096]	[−0.031	…	0.006]
A–C	0.092	0.016	0.063	0.009	0.056	0.006	0.138			0.055			0.484			[−0.011	…	0.069]	[−0.001	…	0.074]	[−0.016	…	0.031]
C–E	0.084	0.006	0.073	0.007	0.063	0.008	0.265			0.071			0.394			[−0.010	…	0.032]	[−0.002	…	0.044]	[−0.015	…	0.034]
A–E	0.119	0.014	0.046	0.009	0.066	0.010	**0.002**	*		**0.013**	*		0.183			[0.035	…	0.110]	[0.014	…	0.092]	[−0.049	…	0.011]
B–D	0.128	0.012	0.116	0.015	0.093	0.007	0.562			**0.030**	*		0.186			[−0.031	…	0.054]	[0.004	…	0.065]	[−0.014	…	0.061]
F–G	0.074	0.017	0.033	0.004	0.027	0.004	**0.045**	*		**0.025**	*		0.266			[0.001	…	0.080]	[0.008	…	0.087]	[−0.006	…	0.019]
G–H	0.099	0.009	0.079	0.008	0.042	0.007	0.127			**0.001**	*		**0.007**	*		[−0.007	…	0.046]	[0.031	…	0.081]	[0.013	…	0.061]
H–I	0.090	0.008	0.062	0.008	0.031	0.005	**0.035**	*		**0.000**	*		**0.010**	*		[0.002	…	0.053]	[0.039	…	0.081]	[0.010	…	0.054]
I–J	0.077	0.011	0.049	0.008	0.030	0.004	0.072			**0.003**	*		0.064			[−0.003	…	0.058]	[0.020	…	0.072]	[−0.001	…	0.039]
F–H	0.067	0.017	0.074	0.007	0.055	0.004	0.726			0.522			**0.050**			[−0.048	…	0.035]	[−0.028	…	0.051]	[0.000	…	0.036]
H–J	0.060	0.013	0.095	0.008	0.052	0.005	**0.048**	*		0.555			**0.002**	*		[−0.069	…	0.000]	[−0.022	…	0.039]	[0.021	…	0.065]
F–J	0.117	0.020	0.051	0.007	0.055	0.012	**0.012**	*		**0.026**	*		0.743			[0.019	…	0.113]	[0.009	…	0.114]	[−0.035	…	0.026]
G–I	0.127	0.012	0.107	0.009	0.072	0.005	0.202			**0.002**	*		**0.007**	*		[−0.013	…	0.053]	[0.027	…	0.084]	[0.012	…	0.058]

**Table 2:** Comparison of the measurements between the three models, for each measurement.

	scanned plaster cast vs plaster cast						scanned impression vs plaster cast							scanned plaster cast vs scanned impression							
measurements	meandifference	95% CI			p-value		meandifference		95% CI			p-value		meandifference		95% CI			p-value		
A–B	−0.385	[−0.420	…	−0.349]	**0.000**	*		−0.360	[−0.426	…	−0.294]	**0.000**	*		−0.024	[−0.088	…	0.039]	0.411		
B–C	−0.545	[−0.606	…	−0.484]	**0.000**	*		−0.354	[−0.429	…	−0.279]	**0.000**	*		−0.191	[−0.293	…	−0.088]	**0.002**	*	
C–D	−0.573	[−0.688	…	−0.459]	**0.000**	*		−0.424	[−0.486	…	−0.361]	**0.000**	*		−0.150	[−0.269	…	−0.031]	**0.019**	*	
D–E	−0.466	[−0.544	…	−0.388]	**0.000**	*		−0.394	[−0.459	…	−0.330]	**0.000**	*		−0.072	[−0.133	…	−0.010]	**0.028**	*	
A–C	0.077	[−0.008	…	0.162]	0.071			0.115	[−0.007	…	0.237]	0.061			−0.038	[−0.100	…	0.023]	0.194		
C–E	0.053	[−0.079	…	0.184]	0.390			0.068	[−0.030	…	0.165]	0.152			−0.015	[−0.182	…	0.151]	0.842		
A–E	0.367	[0.311	…	0.423]	**0.000**	*		0.587	[0.502	…	0.672]	**0.000**	*		−0.220	[−0.308	…	−0.131]	**0.000**	*	
B–D	0.004	[−0.066	…	0.074]	0.900			0.237	[0.183	…	0.291]	**0.000**	*		−0.233	[−0.339	…	−0.128]	**0.001**	*	
F–G	−0.340	[−0.394	…	−0.286]	**0.000**	*		−0.413	[−0.528	…	−0.297]	**0.000**	*		0.073	[−0.044	…	0.189]	0.191		
G–H	−0.579	[−0.649	…	−0.510]	**0.000**	*		−0.457	[−0.632	…	−0.283]	**0.000**	*		−0.122	[−0.265	…	0.022]	0.087		
H–I	−0.420	[−0.487	…	−0.352]	**0.000**	*		−0.352	[−0.455	…	−0.250]	**0.000**	*		−0.067	[−0.124	…	−0.011]	**0.024**	*	
I–J	−0.390	[−0.449	…	−0.311]	**0.000**	*		−0.509	[−0.581	…	−0.437]	**0.000**	*		0.120	[0.070	…	0.170]	**0.000**	*	
F–H	−0.054	[−0.120	…	0.012]	0.096			0.069	[−0.062	…	0.201]	0.265			−0.123	[−0.243	…	−0.003]	**0.045**	*	
H–J	−0.068	[−0.161	…	0.024]	0.128			−0.023	[−0.138	…	0.091]	0.659			−0.045	[−0.170	…	0.079]	0.432		
F–J	0.419	[0.380	…	0.458]	**0.000**	*		0.331	[0.185	…	0.476]	**0.001**	*		0.089	[−0.050	…	0.227]	0.182		
G–I	0.038	[−0.020	…	0.096]	0.175			0.229	[0.120	…	0.338]	**0.001**	*		−0.191	[−0.312	…	−0.070]	**0.006**	*	

As far as the reproducibility was concerned, repeated measurements made on both the scanned plaster casts and the scanned impressions were more reproducible than on the plaster casts. When the measurement error of the plaster casts and scanned plaster casts was compared, nine out of sixteen distances were significantly different. In eight of these cases, the measurement error of the scanned plaster casts was smaller than the measurement error of the plaster casts.

When the measurement error of the plaster casts and scanned impressions were compared, it was found to be significantly different for twelve out of sixteen distances. In all of these cases, the measurement error of the scanned impressions was smaller than the measurement error of the plaster casts.

When the measurement error of the scanned plaster casts and scanned impressions were compared, six distances were significantly different. In all of these cases, the measurement error of the scanned impressions was smaller than the measurement error of the scanned plaster casts.

Even though there were significant differences, all measurement errors were smaller than 0.2 mm, while 81% of all measurement errors were smaller than 0.1 mm.

As far as the accuracy is concerned, when the measurements on the plaster casts were compared to the scanned plaster casts, ten measurements were significantly different. For eight of them, the measurements on the scanned plaster casts were smaller than on the plaster casts.

When the measurements on the plaster casts were compared to the scanned impressions, twelve measurements were significantly different. For eight of them, the measurements on the scanned impressions were smaller than on the plaster casts.

When scanned plaster casts and scanned impressions were compared, nine measurements were significantly different. For eight of them, the measurements on the scanned impressions were smaller than on the scanned plaster casts.

### Analysis of the Color Histograms

**Figure 6:**
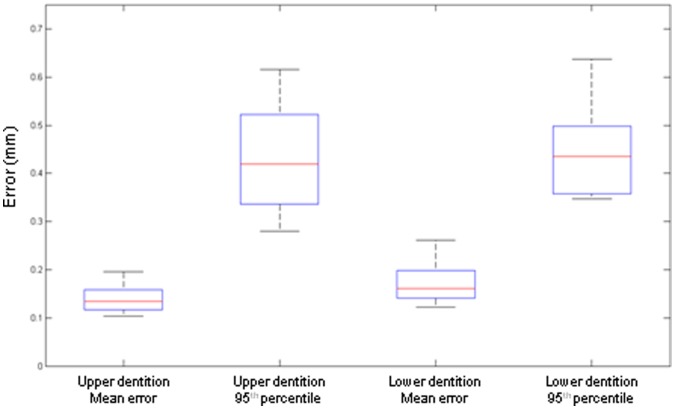
Boxplot of the matched surfaces, showing the mean error and 95^th^ percentile for the upper and lower dentition.

After matching the scanned plaster casts and the scanned impressions, a close relation was seen between the two surfaces. For the upper dental arches a mean error of 0.14 mm (+/−0.14 mm) was found as an indication of the difference between the two surfaces. For the lower dental arches the mean error was 0.18 mm (+/−0.15 mm). To visualize the mean error a box plot was computed (fig. 6).The box plot also shows the 95^th^ percentile of the matching for the 10 models. This shows the range that contains 95% of all corresponding points. In other words: the distance of 95% of all corresponding points is between 0.28 and 0.62 mm for the upper arch and between 0.35 and 0.64 mm for the lower arch (95^th^ percentile, fig. 6).

### Digital Handling Process

The time needed for reconstructing the CBCT scans to a digital patient, where the impressions are integrated into the CBCT scan of the patient takes about 15 minutes, with little variance between patients.

## Discussion

The aim of this study was to evaluate the accuracy and reliability of this new integration method [9], using titanium markers. To do so, we needed to show that the surface of a model, where the markers are attached to, is identical to the surface of the scanned impression. Whether this is a patient, a dry skull or a dental cast, does not matter, since the performed measurements and fusion steps will be the same in all models. What is more, this validation study cannot be performed on patients, since unnecessary radiation exposure should be avoided for medical and ethical reasons. Therefore we randomly selected 10 plaster models, which we handled as if they were patients. After marker placement, these models were scanned, impressions were taken and the impressions were also scanned. This resulted in three models: one physical plaster model (the plaster cast) and two digital models (the scanned plaster cast and the scanned impression). It is without doubt that this is an ex-vivo study that does not fully simulate the clinical condition. A clinical study may show larger errors than those reported here under ideal conditions. We considered the problem of a marker being lost a minor one, because a Procrustes registration is possible with 4 markers as well. However, the present results and small errors were obtained with 5 markers. We do not know if errors would be larger with 4 markers. Also, the amount of error may depend on which specific marker was lost. This should be investigated further.

The results for accuracy and reproducibility show statistically significant differences. However, the 95% confidence intervals (Table 1 and 2) show that most of the ranges are within 0.1 mm, which value can be considered as clinically irrelevant. Furthermore, the mean measurement errors of the digital models are in almost all cases smaller than on plaster casts. This shows that measurements on plaster casts and scanned plaster casts are comparable. This confirms the findings of a recent systematic review on the comparison of measurements on plaster models and digital plaster models [13].

Concerning the accuracy, in general, distances on plaster models were smaller than on digital models. This may be explained partly by the threshold, used in the reconstructions of the markers. The high threshold we chose for extracting the markers ensured that we had no scattering and beam-hardening artifacts in the markers. However, this might have also removed part of the actual markers, making the markers on the digital models smaller, than on the plaster casts.

During the matching procedure, different errors occur, that need to be taken into account. Firstly, during acquisition, small variation between the real object and the captured image is present, resulting in an acquisition error. Due to this, a small error will be present in the reconstructed images of both the scanned plaster cast and the scanned impression. Since locations of these errors will be different for the two scans, a small difference is seen after matching the two surfaces [14], [15]. Secondly, during segmentation some errors occur in reconstructing the markers. Since the voxel size is 0.2 mm, these errors will be very small. Besides that, due to the calibration of the plaster, the segmentation error is kept as small as possible [15], [16]. Finally in the registration process some errors will occur. The marker based matching is actually a Procrustes matching, where the centers of the 5 corresponding markers are matched. Due to the previous mentioned errors, the centers will not completely be the same in the two models, resulting in a small difference [12]. However, since the overall mean error is only 0.14 and 0.18 mm for the upper and lower jaw respectively, this will not be clinically relevant.

Stability of the impression material should also be taken into account. For impressions that need to be shipped to another location, material stability is essential, since scanning or pouring of the impressions is delayed. In this study orthodontic alginate was used. Scanning of the impressions was performed using an in-house scanner within the time advised by the manufacturer. It has been shown when the scanning is performed within the hour, stability is ensured [17].

In most publications describing the previous methods, the time needed for the digital handling processes is not mentioned. Swennen et al. [8] described a total time of 50 minutes for total reconstruction of the patient’s digital face. Choi et al. [6] states that their method takes less than 50 minutes, including scan time. The method proposed in this paper took only 15 minutes which makes the whole digital planning time efficient.

For the present simulation study we used simple industrial superglue (UHU Super Power, methyl-2-cyanoacrylate). This glue however is not suitable for human tissues. Studies have shown that methyl-2-cyanoacrylate provokes acute and chronic tissue reaction and cause histotoxicity due to the exothermic nature of the polymerization reaction of short chain cyanoacrylates. Furthermore, they generate local high concentrations of breakdown products, which include formaldehyde and alkylcyanoacetate [18]. For the clinical procedure, which has been described in detail by Rangel et al 2012 [9], we therefore use a N-butyl 2-cyanoacrylate tissue adhesive (Indermil, Henkel Ireland Ltd., Whitestown, Dublin, Ireland), which is already widely used to close incisions [19], [20]. When used clinically, it is important to instruct the patient not to touch the markers with the tongue when they are glued in place to prevent loosening. It is also a prerequisite to have an in-office CBCT machine to perform scanning of the patient with the markers attached to the gingiva.

Another concern could be the presence of brackets and the scattering that is produced in relation to the position of the markers. Clinically the markers are placed on the attached gingiva, 2 to 3 mm from the gingival margin. This is far enough from the brackets and gives separation from the scatter area.

In conclusion, we can state within the limitations of an ex-vivo study, that this novel method is a reliable method to integrate digital dental casts into CBCT scans. As far as radiation exposure, soft tissue deformation and digital handling processes are concerned, it is a significant improvement compared to the previously published methods. Digital impression taking (intra-oral scanning) will make the procedure even more straightforward. However, the reliability of the latter method needs to be evaluated in a clinical study.
